# The Influences of Ultrasonic Vibrations on Laser Cladding Ni60/WC-TiO_2_+La_2_O_3_ Composite Coating

**DOI:** 10.3390/ma16196356

**Published:** 2023-09-22

**Authors:** Xu Huang, Yanchun Chen, Jibin Jiang, Guofu Lian, Changrong Chen

**Affiliations:** 1 Fujian Key Laboratory of Intelligent Machining Technology and Equipment, Fujian University of Technology, Fuzhou 350118, China; huangxu@fjut.edu.cn (X.H.); gflian@mail.ustc.edu.cn (G.L.); 2 Department of Mechanics, School of Mechanical and Automotive Engineering, Fujian University of Technology, Fuzhou 350118, China; chenyc19980102@163.com (Y.C.); changrong.chen@fjut.edu.cn (C.C.)

**Keywords:** laser cladding, ultrasound, vibration frequency, rare earth oxide, cladded powders

## Abstract

The optimal process parameters of ultrasonic-assisted processing were studied to further improve the molding quality and mechanical properties of Ni60/WC-TiO_2_+La_2_O_3_ composite coating. A single-factor experiment was used to explore the influences of ultrasonic vibration frequencies on Ni60/WC-TiO_2_+La_2_O_3_ composite coating. The microstructure, elemental composition, phase composition, hardness, and wear resistance of the coating were studied using scanning electron microscopy (SEM), an X-ray diffractometer (XRD), an energy spectrometer, a microhardness meter, a friction and wear tester, and other equipment. Ultrasonic vibrations significantly improved the problems of pores in the coating, and the porosity was reduced from 0.13 to 0.014%. When the vibration frequency was 32 kHz in the experiment, the aspect ratio of the coating was optimized from 2.06 to 2.48, the dilution rate increased from 5.60 to 5.79%, the hardness increased from 960.25 to 988.45 HZ_1.0_, and the friction coefficient was reduced from 0.34 to 0.27. The coating performance was significantly improved, and the research results provide a reference for preparing excellent Ni60/WC-TiC+La_2_O_3_ composite coating.

## 1. Introduction

Laser cladding uses laser beams with high energy density to melt powders. A dense and good metallurgical coating [1,2,3] is formed with the substrate surface to complete the surface modification. A coating with a low dilution rate and low thermal deformation is widely used in metal surface modification and the repair of parts [4,5]. Recently, auxiliary processing has become one of the current research hotspots with the development of laser cladding. Ultrasonic vibration is used as an auxiliary means of laser forming, and its action principles on the molten pool include the cavitation effect [6], acoustic-streaming effect [7], and thermal effect [8]. It has shown great potential in reducing the porosity as well as optimizing the aspect ratio and mechanical properties [9,10].

Ma et al. [11] applied ultrasonic vibrations during the laser cladding process to improve the defects of pores in the coating. Yttria-stabilized zirconia (YSZ) powder coatings are prepared on a titanium alloy. The coating grains exhibit finer morphology following the application of ultrasonic vibrations, and they predominantly comprise fine isometric crystals with a minor presence of dendritic structures. The porosity and crack defects were significantly reduced, which improves hardness and wear resistance. Zhang et al. [12] prepared the TiB_2_-TiC ceramic-particle-reinforced iron-based composite coating through the ultrasonic-vibration-assisted laser cladding process. The surface morphology of the coating is smoother after the ultrasonic vibration is applied. As the ultrasonic vibration power increases, the dilution rate of the coating increases due to the combined effects of cavitation, acoustic streaming, and mechanical vibrations. Ultrasonic vibrations increase the heat transfer efficiency of the coating. The decomposition of ceramic-reinforced particles increases with more uniform distribution, which refines the grain structure of the molten pool. When the ultrasonic vibration power is 300 W and the frequency is 20 kHz, the coating has the least pores and cracks. Li et al. [13] prepared a Ni-WC-CaF_2_ coating using an ultrasonic-vibration-assisted laser cladding process. Ultrasonic vibrations can reduce the aggregation degree of WC particles during the laser cladding process. Moreover, it improves the crystal grain organization at the interface between the coating and the substrate and refines crystal grains. The hardness and wear resistance of the coating are optimal at the ultrasonic vibration power of 900 W and 20 kHz. The above research shows the significant influence of ultrasound on coating morphology. However, current research on its influence law and mechanism is relatively limited, and further in-depth research is required.

This work studied the microstructure of composite Ni60/WC@TiO_2_+La_2_O_3_ and the special conditions of the molten pool of the composite at high temperatures. We investigated the influence of the ultrasonic vibration frequency on the microstructure and mechanical properties of the Ni60/WC@TiO_2_+La_2_O_3_ composite coating as well as its underlying mechanisms. Additionally, auxiliary processing techniques were explored to optimize the composite coating.

## 2. Materials and Methods

### 2.1. Sample Preparation

This work used 45 steel (40 × 20 × 10 mm) as the substrate (Table 1). The cladding material was a powder material with 59% Ni60 + 40% WC-TiO_2_ + 1% La_2_O_3_ added (see Table 2 for the chemical composition of Ni60). A single-factor experimental method was used.

### 2.2. Experimental Process

The Stober method was used in the test, with polyvinylpyrrolidone (PVP) as the coupling agent, tetrabutyl titanate as the precursor of TiO_2_, and ammonia and deionized water as the catalysts. They were coated with absolute ethyl alcohol to obtain WC-TiO_2_ powders with a surface covering the TiO_2_ shell. The powders were mixed with La_2_O_3_ and dried for use.

The oily surface of the substrate was washed with absolute ethanol before cladding. A laser cladding test was carried out with a fiber laser (YLS-3000, IPG Photonics, Oxford, MA, USA). According to the literature, the process parameters for cladding were set as follows: a laser power of 1800 W, a defocusing amount of 10 mm, and a scanning speed of 5 mm/s. All samples were single-channel cladding; argon was used as a protective gas with its oxygen content not exceeding 1%. Ultrasonic vibrations were applied during the laser cladding process, the ultrasonic vibration source was positioned at the bottom of the sample, and the coating was primarily influenced by longitudinal waves and 6 sets of single-factor tests were carried out by changing the ultrasonic vibration frequency. Table 3 presents the specific test plan.

### 2.3. Characterization

**Figure 1:**
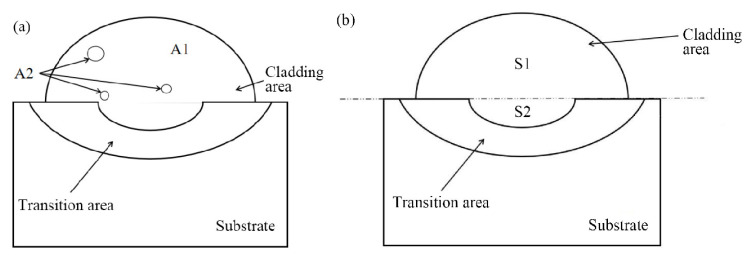
Porosity (**a**) and dilution rate (**b**) calculation schematic.

The porosity and dilution rate of samples are determined using the area method with Equations (1) and (2), respectively (Figure 1a,b). ImageJ software is employed for data measurement.

(1) $$\begin{array}{l} w=\sum A_{2}/A_{1}*100\% \end{array}$$

*D* = *S*2/*S*1 + *S*2 (2)

*A*_1_ is the total area of the coating; *A*_2_ is the pore area; *D* is the dilution rate of the coating; *S*1 is the area of the coating’s stacking zone; and *S*2 is the area of the matrix melting zone.

The microstructure of the coating was observed using a scanning electron microscope (SEM), while the compositions of its microregions were analyzed using an energy-dispersive X-ray spectrometer (EDS). Microhardness measurements were conducted from the top of the coating to the substrate. The measurement employed a microhardness tester with a load of 9.81 N and the dwell time of 20 s. Tests were performed three times in nearby regions with the average value taken.

Surface roughness was controlled through grinding and polishing processes before friction wear experiments, followed by cleaning with anhydrous ethanol. Subsequently, wear resistance testing was carried out using a friction wear test machine at room temperature. Dry friction experiments were conducted utilizing tungsten steel balls with a diameter of 10 mm as abrasive materials. The motion pattern involved reciprocating linear movements with a distance of 4 mm at a load of 25 N. Each sample was tested for 60 min. Sample surfaces were cleaned again with anhydrous ethanol after the friction wear experiments. Then, the 3D morphology and volume of worn regions were observed and calculated using white light interferometry.

## 3. Results and Discussion

### 3.1. Macrostructure of the Cladding Layer

**Figure 2:**
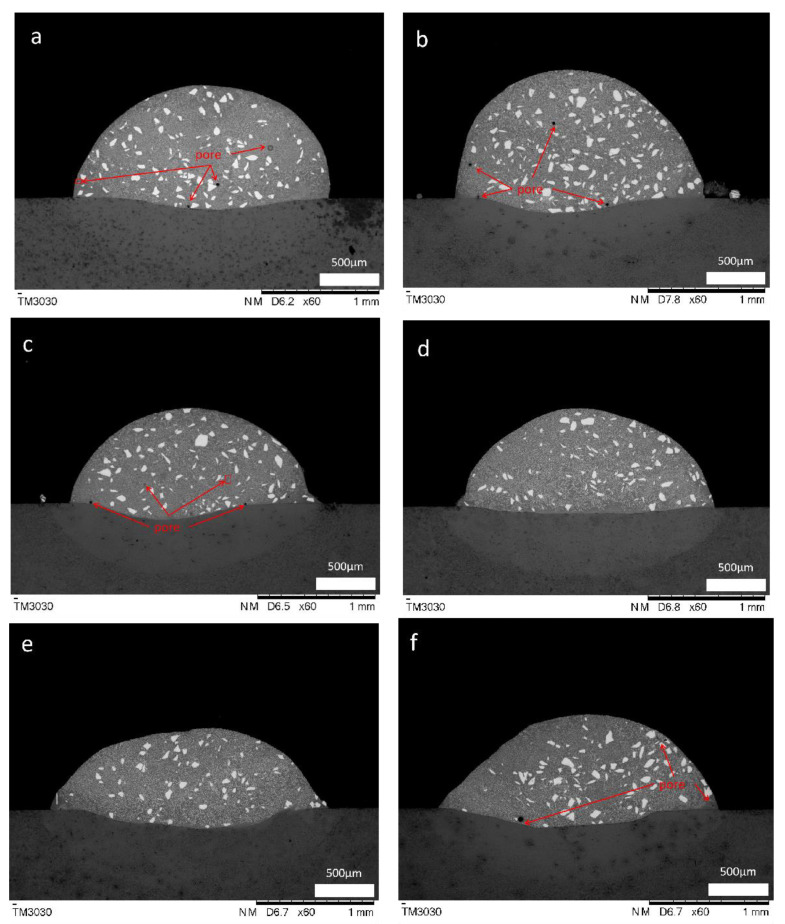
Cross-sectional morphology of the cladding layer with different ultrasonic frequencies applied: (**a**) 0 kHz, (**b**) 22 kHz, (**c**) 27 kHz, (**d**) 32 kHz, (**e**) 37 kHz, (**f**) 42 kHz.

Figure 2 shows the cross-sectional morphology of the coating with different ultrasonic frequencies applied. A larger pore and two smaller pores exist in the cross-section of the coating without ultrasonic vibrations. The pore size of the coating becomes smaller but the number increases after ultrasonic vibrations are applied (Figure 2b,c). The pores are mainly concentrated on the outside of the coating, and dilution occurs at the junction between the coating and the substrate (Figure 2f). As the ultrasonic vibration frequency increases, the morphology of the fusion zone of the coating changes. The influence of ultrasonic vibrations on the coating is analyzed through the changes in the porosity, dilution rate, morphology of the fusion zone, and aspect ratio of the coating.

### 3.2. Layer Porosity Analysis

**Figure 3:**
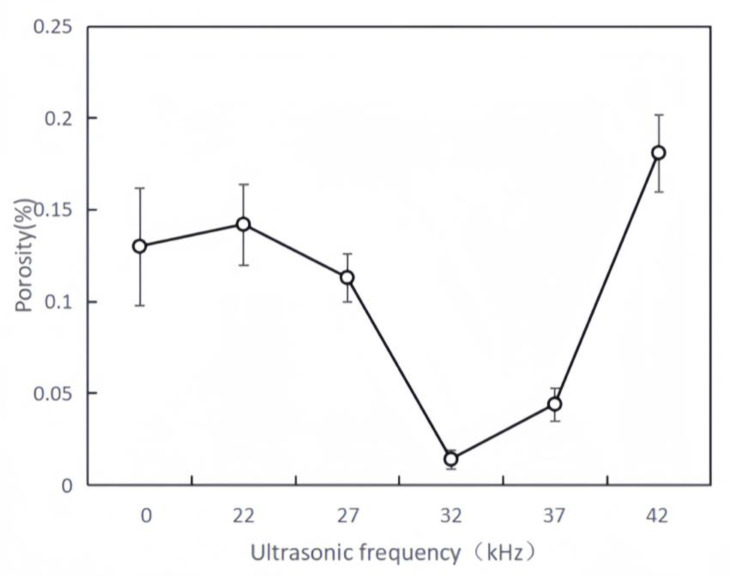
Statistical chart depicting the scattering of porosity in the coating.

Table 4 lists the calculated porosity and its corresponding standard deviations. The porosity of the coating changes regularly with the changes in the ultrasonic vibration frequency (Figure 3). When ultrasonic vibrations are not applied to the coating with rare earth added, four obvious pores exist.

When the ultrasonic vibration frequency is 32 kHz, there are no pores in the coating. As the ultrasonic vibration frequency increases, the porosity decreases first and then rises. When the ultrasonic vibration frequency is 22 or 27 kHz, the porosity of the coating does not change significantly. The elimination mechanism of ultrasonic vibrations on the pores of the coating is mainly the cavitation effect. The ultrasonic sound intensity required for the rupture of the cavitation bubbles is affected by the structural strength of the metal melt and the intermolecular force [14]. Therefore, a cavitation threshold exists. The cavitation threshold is related to the boiling point, ambient temperature, and viscosity of the metal melt.

The powders in this test were added with 40% of WC-TiO_2_ and rare earth powders, which are both high-melting-point materials. Therefore, the cavitation threshold is higher. When the ultrasonic vibration frequency is 22 or 27 kHz, the ultrasonic sound intensity does not exceed the cavitation threshold. The cavitation effect is weak, with no significant change in the porosity. A significant cavitation effect occurs in the coating until the ultrasonic vibration frequency reaches 32 kHz. Combined with the sound flow strengthening effect, the convection effect is enhanced in the molten pool, so the air bubbles quickly float to the surface and escape.

When the ultrasonic vibration frequency increases, the application of high-frequency ultrasonic vibrations induces the rapid pulsating oscillations of liquid metals within the molten pool. Therefore, the stability and surface tension of the molten pool are affected. As a result of the vibrational effect, unstable surface ripples and bulges are prone to form during solidification. Ripples and bulges increase gas inclusions, with pores generated throughout the solidification process. The air bubbles that escape from the molten pool before solidification form pores (Figure 2f) because of the fast melting and rapid condensation of laser cladding. Some pores are too late to surface on the right side of the coating.

The porosity of the conventional nickel-based coating typically ranges from 2.1 to 3.2% after laser cladding. The porosity of the coating formed through the experiment is lower than that of the conventional nickel-based coating [15]. The coating prepared in the experiment achieved a superior level and underwent optimization.

### 3.3. Analysis of the Dilution Rate and Aspect Ratio

**Figure 4:**
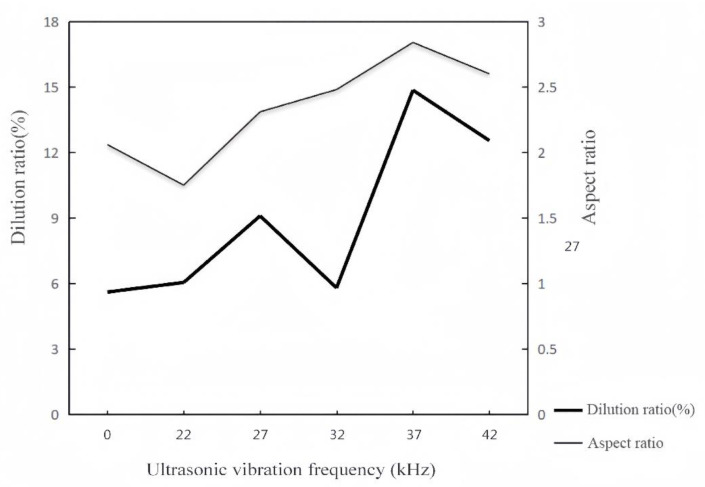
Dilution rate of fused cladding layers and their aspect ratios with different ultrasonic frequencies applied.

As the ultrasonic vibration frequency increases, the coating height decreases and the width increases (Figure 2). These changes can be characterized by the aspect ratio: the higher the aspect ratio value, the flatter the overall morphology of the coating. The aspect ratio and dilution rate of the coating are calculated, and Table 5 presents the results. Origin software is used to plot the measurement results (Figure 4).

The aspect ratio of the coating gradually increases (Figure 4) due to the thermal effect of ultrasonic-vibration-assisted processing with the increased ultrasonic vibration frequency. The thermal effect produced by the cavitation effect and the acoustic-streaming effect strengthens the temperature and internal flow speed of the molten pool, which causes the molten pool to flow more to both sides. The height of the coating is reduced and the width is increased [16].

**Figure 5:**
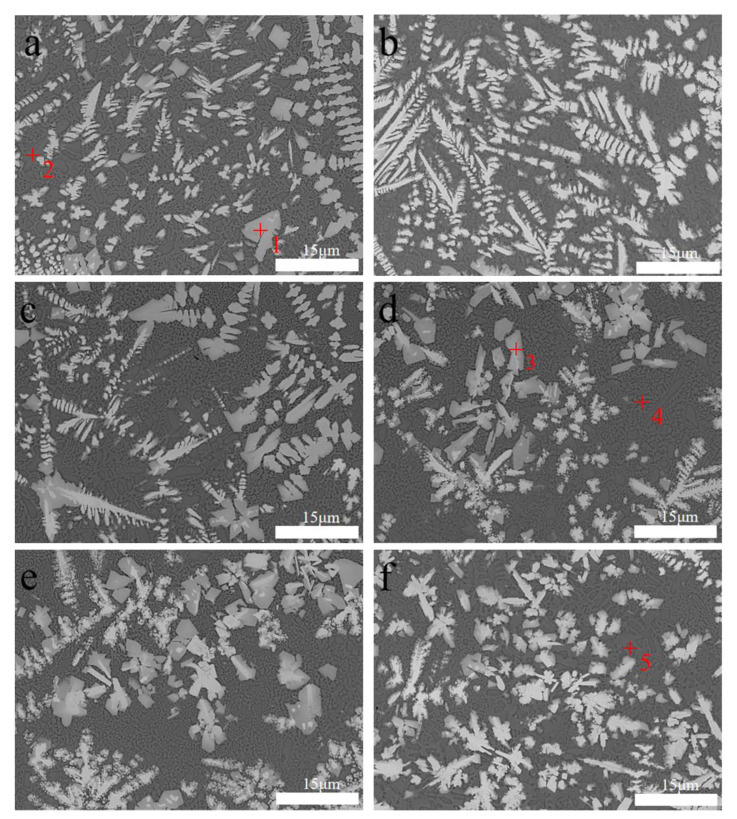
Microstructure of the middle part of the cladding layer subjected to different ultrasonic frequencies: (**a**) 0 kHz, (**b**) 22 kHz, (**c**) 27 kHz, (**d**) 32 kHz, (**e**) 37 kHz, (**f**) 42 kHz.

When the ultrasonic vibration frequency is 22 kHz, the dilution rate of the coating does not change much, and the cavitation effect and the sound flow effect are not obvious (Figure 5). When the frequency increases to 27 kHz, the dilution rate increases. The sound flow effect is enhanced, and the energy in the molten pool increases. When the frequency increases to 32 kHz, the dilution rate decreases. A strong cavitation effect is produced, and the fluidity of the molten pool is enhanced. The molten pool flows more to both sides, which increases the contact area with the substrate [17]. Therefore, the depth of the molten matrix decreases with the decreased dilution rate.

When the ultrasonic vibration frequency continues to increase, the acoustic-streaming effect and the thermal effect dominate. The convection of the molten pool intensifies, and the friction generated by convection increases the temperature. It accelerates the dissolution of the powder particles of the coating and eliminates the deposition effect of some larger particles [18]. Therefore, the dilution rate increases to more than 12%, and the morphology of the fusion zone changes.

### 3.4. Microstructure Analysis

**Figure 6:**
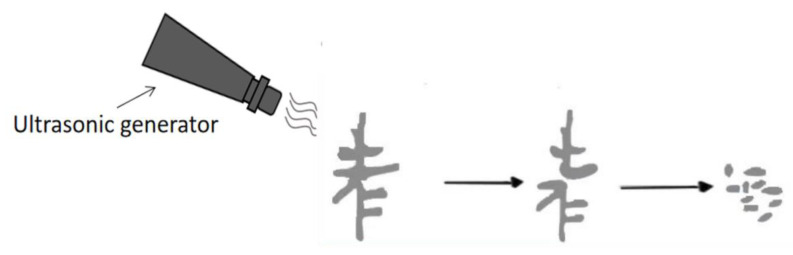
Schematic diagram of the action of ultrasonic vibration.

Figure 6 shows the microstructure of the middle part of the coating with different ultrasonic frequencies applied; Table 6 presents the corresponding EDS analysis results. When the ultrasonic vibration is not applied and the ultrasonic vibration frequencies are 22 and 27 kHz, slender needle crystals and dendrites exist in the coating. The coating at 22 kHz is affected by the longitudinal mechanical wave of the ultrasonic vibration and the temperature gradient. Moreover, the growth direction of the dendrites in the coating tends to be inclined toward the top. Sharp needle crystals and dendrites still exist at 27 kHz; however, the randomness of the growth direction increases. As the frequency increases, the acoustic-streaming effect increases. The molten pool is affected by the acoustic-streaming effect and the thermal effect, and fluidity is stronger than that at 22 kHz. The dendrites affected by the longitudinal wave deviate from the original growth direction with the flow of the molten pool.

According to the statistics, the average grain sizes of the main metallographic phases of M_23_C_6_ and Ni-Cr-Fe of each coating are 0.307, 0.296, 0.288, 0.201, 0.243, and 0.244 µm, respectively. The grain sizes of the coatings show little difference with/without ultrasonic vibrations at 22 and 27 kHz because the critical value for the cavitation effect has not yet been reached.

When the ultrasonic vibration frequency reaches 32 kHz, the needle crystals and slender dendrites in the coating are significantly reduced, while the isometric crystals increase. A cavitation effect occurs, and the rupture of the cavitation bubbles with the ultrasonic cavitation effect affects the molten pool. The growing robust dendrites break (Figure 6) and are evenly distributed in the molten pool under the pull of turbulence and circulation caused by the acoustic-streaming effect [19]. Meanwhile, the enhanced acoustic-streaming effect and thermal effect and the increased fluidity of the molten pool inhibit the growth rate of dendrites and refine the grains.

When the ultrasonic vibration frequency is 37 or 42 KHz, the grain size increases. Combined with points P4 and P5 in the EDS analysis, the weakened cavitation effect and the excessive thermal effect increase the dilution rate of the coating. Excessive 45 steel substrates melt into the molten pool.

### 3.5. Microhardness Analysis

**Figure 7:**
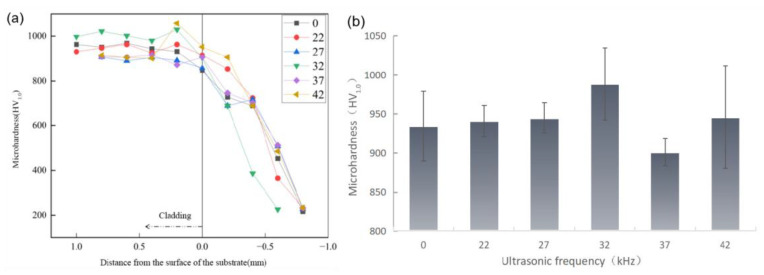
Line graph of microhardness of cladding layer (**a**) and statistical chart depicting the scattering of microhardness in the coating (**b**).

Figure 7 shows the microhardness of the coating. The microhardness of the coating with/without ultrasonic vibrations is unchanged at 22 kHz. No cavitation effect is produced, and the intensity of the acoustic-streaming and thermal effects is not high, which weakens the coating. The microhardness of the coating decreases at 27 kHz. The threshold of the cavitation effect has not yet been reached. The leading roles in the coating are the acoustic-streaming effect and the thermal effect.

The decomposition of WC particles increases. More C and O enter the molten pool, which forms CO/CO_2_ bubbles and large particles of hard and brittle carbides. Air bubbles are crushed into small air bubbles [19]. They fail to escape from the molten pool and solidify into pore defects, which affect coating hardness. Large particles of hard and brittle carbides can also affect hardness.

When the ultrasonic vibration frequency is 32 kHz, the average microhardness of the coating is 988.45 HZ1.0. The average microhardness of the coating is the highest, and coating hardness is increased by 5.77% compared to that without ultrasonic vibrations. Based on the microstructure analysis, the grain refinement of the coating affected by the cavitation effect increases the microhardness of the coating. Although there are more thermal effects, the acoustic-streaming strengthening effect accelerates the flow of the molten pool. A faster flow rate can diverge heat through the substrate and the upper surface of the molten pool as well as promote grain refinement.

When the ultrasonic vibration frequencies are 37 and 42 KHz, a decrease in the microhardness of the coating can be observed. The internal temperature and flow rate of the molten pool increase with the increased acoustic-streaming effect and thermal effect. More substrate materials enter the molten pool, which increases impurities and affects the microhardness of the coating.

### 3.6. Analysis of Frictions and Wear Properties

**Figure 8:**
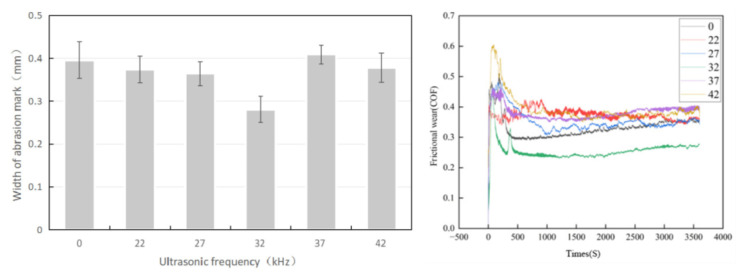
Friction coefficient and abrasion width of fused cladding layers with different ultrasonic vibration frequencies.

Figure 8 presents the friction trace width and the friction coefficient of the coating with different ultrasonic vibration frequencies applied. The wear resistance of the coating changes little at 22 and 27 kHz compared to that without the ultrasonic vibration. The cavitation effect caused by ultrasonic vibrations is weak, and the molten pool with 1.0% La_2_O_3_ addition after using TiO_2_ to coat WC particles has higher fluidity. The acoustic-streaming effect on the molten pool is relatively weak, and the friction coefficient does not change much.

When the applied ultrasonic vibration frequency is 32 kHz, the friction coefficient and wear scar width of the coating are significantly reduced, indicating a significant improvement in the wear resistance of the coating. The friction coefficient is 0.27, which is 23.85% lower than that without the ultrasonic vibration; the wear scar width is 0.281 mm. Combined with macroscopic morphology and microstructure analysis, the cavitation effect in the coating is enhanced. The instantaneous high temperature and high pressure are generated by the collapse of the cavitation nuclei. Growing dendrite grains are torn to refine the grains, which strengthens the wear resistance of the coating.

When the ultrasonic vibration frequencies are 37 and 42 KHz, the cavitation effect weakens as the cavitation threshold increases. The acoustic-streaming effect and the thermal effect dominate. The thermal effect increases the temperature of the molten pool and the number of WC particles dissolved, which results in more carbon monoxide or carbon dioxide bubbles [20]. The air bubbles that do not escape from the molten pool form pore defects, which reduces the wear resistance of the coating. Moreover, the acoustic-streaming effect further enhances the fluidity of the molten pool, so the molten pool with higher temperatures can melt more substrate materials. Impurities enter the molten pool, which reduces the wear resistance of the coating.

**Figure 9:**
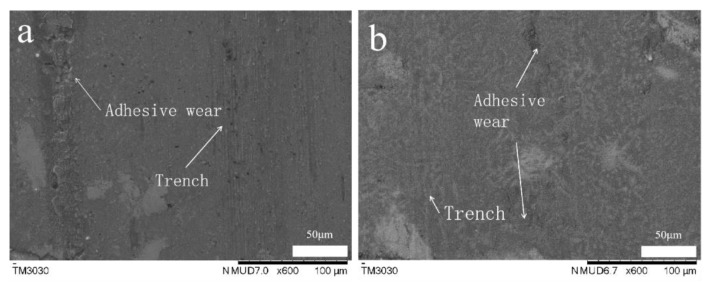
SEM photographs of abrasion marks on the fused cladding layer with different ultrasonic vibration frequencies applied: (**a**) 0 kHz and (**b**) 32 kHz.

Figure 9 shows the SEM of the wear scars of the coating with/without the ultrasonic vibration (32 kHz). When the ultrasonic vibration is not applied, large adhesive wear and peeling pits exist on the wear scars of the coating. Metal adhesion occurs between the friction pair and the coating during the sliding friction process. The adhesion points are sheared and broken during relative sliding, which transfers materials. Therefore, there are peeling pits and metal grinding chips on the surface of the wear scars. The wear mechanism of the coating corresponds to severe adhesive wear; the flaking pits of adhesive wear become shallow and small after ultrasonic vibrations at 32 kHz. Meanwhile, the wear scar grooves are significantly shallower than those in Figure 9a. Combined with microstructure analysis and microhardness analysis, the grain structure of the coating is refined with improved hardness; therefore, the friction properties are better.

## 4. Conclusions

This study tested the laser cladding nickel-based composite coating optimized by rare earth oxides. Ultrasonic-vibration-assisted processing technology was used to further optimize the hardness and wear friction properties of the coating. The ultrasonic vibration power was determined based on the current research status and content. Then, this work investigated the effects of varying ultrasonic vibration frequencies on the microstructure and properties of the coating as well as the mechanism of ultrasonic vibrations on the coating.

(1) As the ultrasonic vibration frequency increased, the porosity first decreased and then increased with the increased aspect ratio. When the ultrasonic vibration frequency reached 32 kHz, the needle crystals and slender dendrites in the coating were significantly reduced, and the isometric crystals increased. The average grain size was reduced to about 0.201 µm, indicating the significant grain refinement effect of the coating.

(2) When the vibration frequency reaches the cavitation threshold, the convection effect in the molten pool was enhanced when combined with the acoustic-streaming effect. The air bubbles in the molten pool quickly floated to the surface and escaped, which decreased the porosity. Meanwhile, the rupture of the cavitation bubbles with the ultrasonic cavitation effect affected the molten pool. The growing robust dendrites broke, which refined the grains.

(3) When the ultrasonic vibration frequency was 32 kHz, the porosity of the coating was the lowest (0.014%) in the experimental group. Coating hardness was the highest (988.45 HZ1.0), which increased by 5.77% compared to that without ultrasonic vibrations. The frictional wear coefficient of the coating was the smallest (0.27), which was 23.85% lower than that without ultrasonic vibrations. Therefore, the best ultrasonic vibration frequency is 32 kHz for the coating.

## Figures and Tables

**Table 1:** Forty-five steel content (mass fraction).

Element	C	Si	Ni	Cr	Mn	Cu	Fe
Proportion	0.42~0.50	0.17~0.37	≤0.30	≤0.25	0.50~0.80	≤0.25	Ba1

**Table 2:** Elemental composition of Ni60 powders.

Element	C	Cr	B	Si	Fe	Ni
Proportion	0.9	16	3.2	4	≤5	Ba1

**Table 3:** Test parameters of the ultrasonic generator.

Power (W)	Test Number	Ultrasonic Frequency (kHz)
	e1	0
	e2	22
540	e3	27
	e4	32
	e5	37
	e6	42

**Table 4:** Porosity and its corresponding standard deviation calculation results.

Ultrasonic Vibration Frequency	Porosity (%)	Standard Deviation
0	0.130	0.032
22	0.142	0.022
27	0.113	0.013
32	0.014	0.005
37	0.044	0.009
42	0.181	0.021

**Table 5:** Calculation results of the aspect ratio and dilution rate of the coating.

	Test Sample	Ultrasonic Vibration Frequency (kHz)					
Calculation Results		0	22	27	32	37	42
Aspect ratio		2.06	1.75	2.31	2.48	2.84	2.60
Dilution rate (%)		5.60	6.04	9.09	5.79	14.85	12.54

**Table 6:** EDS results in the middle of the coating.(P1–P5 respectively correspond to the red numbers in Figure 5).

Number	Chemical Composition (At.%)						
	Ni	Cr	Fe	W	C	La	Ti
**P1**	18.441	43.617	2.325	11.318	24.299	0	0
**P2**	73.664	9.261	4.641	0	9.841	1.364	1.229
**P3**	29.51	41.069	2.337	9.112	17.841	0.131	0
**P4**	57.187	10.116	5.417	4.911	10.839	5.51	6.02
**P5**	65.826	11.42	8.994	0	10.078	1.482	2.2

## Data Availability

Not applicable.
